# Knowledge deficits and barriers to performing soft-tissue coverage procedures: An analysis of participants in an orthopaedic surgical skills training course in Mexico

**DOI:** 10.1097/OI9.0000000000000044

**Published:** 2019-12-20

**Authors:** Patrick D. Albright, Madeline C. Mackechnie, J. Hunter Jackson, Aman Chopra, Jordan T. Holler, Antonio Flores Biard, Luis G. Padilla Rojas, Saam Morshed, Theodore Miclau, David W. Shearer, Michael J. Terry

**Affiliations:** a Institute for Global Orthopaedics and Traumatology, University of California, San Francisco, CA; b Hospital Ángeles del Carmen, Guadalajara; c Centro Médico Puerta de Hierro Especialidades, Zapopan, Mexico

**Keywords:** barriers, flaps, Latin America, orthopaedics, soft-tissue management, surgical training course

## Abstract

**Background:**

An increasing number of traumatic injuries in low- and low-middle-income countries (LICs/LMICs) have coexisting injuries requiring soft-tissue coverage (flaps). Yet, there is a lack of subspecialty care and flap training in Latin America. This study assesses the effectiveness of a surgical skills training course in improving rotational and free flap knowledge and identifies barriers to performing these types of flaps.

**Methods:**

Participants attending a surgical skills training course in Guadalajara, Mexico completed a pre/postcourse flaps knowledge survey consisting of 15 questions from the plastic surgery in-training examination and also completed a 7-point Likert survey regarding perceived barriers to performing flaps at their institution.

**Results:**

Of the course participants, 17 (44.7%) completed the precourse knowledge survey, 24 (63.2%) completed the postcourse survey, and 37 (97.4%) completed the barriers survey. Scores improved from pre- to postcourse knowledge surveys (39.6% to 53.6%, *P* = .005). Plastic surgery subsection scores also improved (39.0% to 60.4%, *P* = .003). Twenty-five percent of attendees received prior flap training and had plastic surgeons available to perform flaps. Few participants (38.9%) reported flap procedures being commonly completed at their hospitals. Participants stating that flaps were uncommon in their hospital reported more institutional barriers and less access to dermatomes. These participants also reported lack of operating room and surgical personnel availability.

**Conclusion:**

A surgical skills training course may be useful in improving knowledge of soft-tissue coverage procedures. There are also modifiable physician and institutional barriers that can improve the ability to perform rotational and free flaps as identified by the course participants.

## 1 Introduction

Musculoskeletal injuries are a major cause of global morbidity and mortality, with rates of extremity injuries in low- and low-middle-income countries (LMICs) exceeding those of high-income countries (HICs) 2 to 5-fold.^[1,2]^ Lower extremity fractures are among the top nonfatal injuries sustained globally, and a significant amount of these injuries in LMICs present with soft-tissue injuries requiring muscle flaps or skin grafts.^[3,4]^ Further adding to the burden of disease, despite the status of many Latin American countries as middle- to upper-middle income countries, there is still an unequal distribution of wealth in these countries, predisposing a significant proportion of their populations to medical conditions normally found in countries with lower GDPs.^[5]^ Failure to properly manage soft-tissue injuries leads to an increased risk of infection and amputation, while implementation of appropriate soft-tissue management allows for adequate wound closure and reduced rates of nonunion.^[6]^

Multidisciplinary treatment is often required for successful management of fractures with accompanying soft-tissue defects, but LMICs face limited access to specialty care.^[1,7]^ For this reason, there exists a strong need for more training, particularly as it pertains to soft-tissue coverage techniques. Knowledge exchange and training courses conducted by HIC specialists who can teach local professionals is a suggested method to build surgical capacity.^[8–10]^ Training local orthopaedic surgeons in locations where there is little surgical subspecialist care may be a solution to promote complex soft-tissue defect management in LMICs,^[11–13]^ and the Surgical Management and Reconstructive Training (SMART) course is one such approach that teaches orthopaedic surgeons the principles of soft-tissue reconstruction and complex fracture management.^[10]^ The curriculum emphasizes lower extremity soft-tissue coverage (flaps) that can be performed without microvascular surgery.^[4,14]^ A follow-up study of a recent SMART course showed that course attendees report increased confidence in and competency of plastic surgery techniques when performing muscle flaps, as well as a 93% self-reported success rate of flap surgeries performed postcourse.^[4,15]^

Despite SMART courses delivering potentially promising outcomes, many participants from LMICs have difficulty applying the course concepts in actual practice. Local resource constraints and pedagogical issues, among other barriers, may be responsible for attendees unsuccessfully implementing their newly acquired knowledge. A SMART course was recently completed in Mexico, a country in which the open tibia fracture annual incidence may be as high as 50,000 per year^[16]^ for surgeons in Latin America. Based on prior studies, we hypothesized that the SMART course would aid in knowledge acquisition and retention in a cohort of Latin American surgeons and that multiple barriers exist to performing flaps including resource limitations and lack of extensive surgical training. We thus completed a survey of SMART course participants in order to assess knowledge acquisition and to identify barriers to performing soft-tissue reconstruction.

## 2 Methods

The inaugural 2019 SMART course in Latin America was similar to prior courses in Nepal, Tanzania, and San Francisco and consisted of didactics, case-based discussions, and video review of cadaver dissections.^[4,14,15,17]^ This study was supported by the Federación Mexicana de Colegios de Ortopedia y Traumatología (FEMECOT) and approved as exempt by the institutional review board of the University of California, San Francisco.

### 2.1 Soft-tissue coverage knowledge survey

A soft-tissue coverage knowledge survey was developed from retired United States plastic surgery in-training examination questions with the input of a fellowship trained plastic surgeon and 2 trauma fellowship trained orthopaedic surgeons. The survey consisted of 15 questions, with half of the questions pertaining to plastic surgery and lower extremity flap procedures, and the other half pertaining to orthopaedic surgery and management of lower extremity open fractures with soft-tissue defects. The survey was translated into Spanish and physical copies were provided to the participants for the pre- and postcourse assessment.

### 2.2 Barriers to performing soft-tissue coverage procedures survey

Investigators developed a survey based on the existing literature^[18–20]^ and the expert opinions of SMART course faculty and local FEMECOT partners. The survey utilized a 7-point Likert scale to assess participant familiarity with flaps, performance of these procedures in their hospital, confidence in performing flaps, and physician, institution, and patient barriers to carrying out flaps. The Likert scale was 7-points (1 = Strongly Disagree, 2 = Disagree, 3 = Slightly Disagree, 4 = Neutral, 5 = Slightly Agree, 6 = Agree, 7 = Strongly Agree). The survey was also translated into Spanish and completed by course participants during the 4-day course. Participant subgroups were identified a priori to assess confidence and barriers to performing flap procedures. These groups included participants having received previous flap training, those stating that flaps were commonly performed in their hospital, and those stating that their prior training was adequate.

### 2.3 Statistical analysis

Descriptive statistics are reported to summarize participant knowledge survey and barriers survey responses. An unpaired, one-sided Student *t* test was used for assessment of average knowledge score between pre- and posttest groups. For the flap barriers survey, an unpaired, two-sided Student *t* test was used for assessment of continuous data and Pearson's Chi-Squared test for categorical data. All data analysis was completed using STATA SE v15.0 (STATACorp, College Station, TX) with significance set to *P* < .05.

## 3 Results

The SMART course consisted of 38 participants from 3 Latin American countries with the majority from Mexico (89.9%) and the others from Cuba (5.6%) and Venezuela (5.6%). No participant had previously attended an SMART course. The majority were male (94.6%), orthopaedic surgeons (91.2%), and fellowship trained in orthopaedics and musculoskeletal trauma (62.2%). Few participants (25%) stated that plastic surgeons are readily available to perform flaps in their hospital, and few (25%) had received any prior flap training. Flaps were reported as uncommonly performed in most hospitals (61.1%). While the majority of participants do not have dermatomes readily available (66.7%), the majority reported access to humby knives (88.2%). Participants most commonly cited physician (39.4%) or institutional issues (36.4%) as being the most important barriers (Table 1).

**Table 1:**
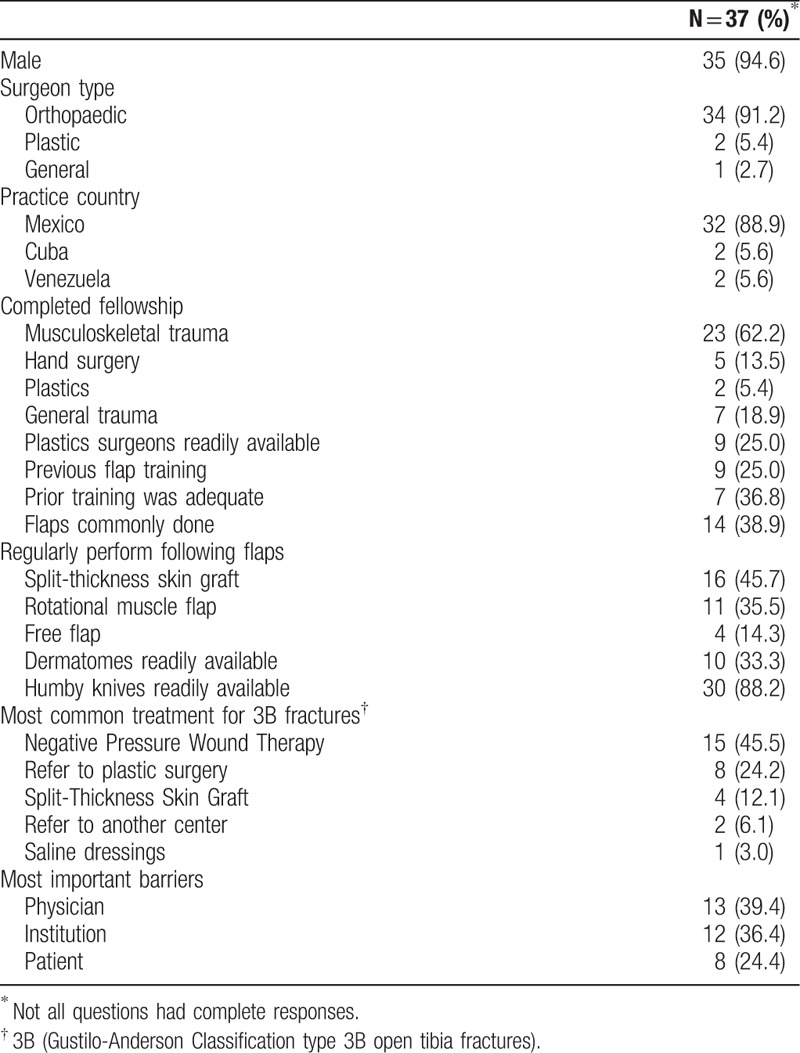
Course participant characteristics

### 3.1 Knowledge survey results

**Figure 1:**
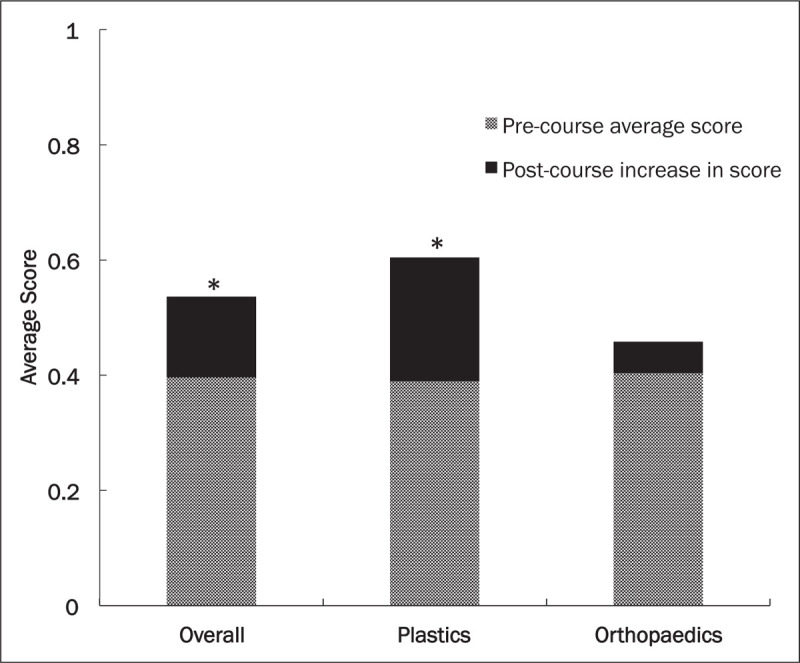
Average overall knowledge survey scores with average score in plastics and orthopaedics subdomains before the SMART course and increase in postcourse average score. ^∗^Statistically significant increase in average score, *P* < .05.

Overall, participants performed significantly better on the postcourse survey compared with the precourse survey, with scores improving from 39.6% ± 15.9 to 53.6% ± 17.1 (*P* = .005). Participants also performed significantly better on the plastics component of the survey, with precourse and postcourse scores improving from 39.0% ± 22.5 to 60.4% ± 24.4 (*P* = .003). Participants improved on the orthopaedics component of the survey from 40.3% ± 15.3% to 45.8% ± 22.2% (*P* = .1777) (Fig. 1).

### 3.2 Barriers survey results

**Figure 2:**
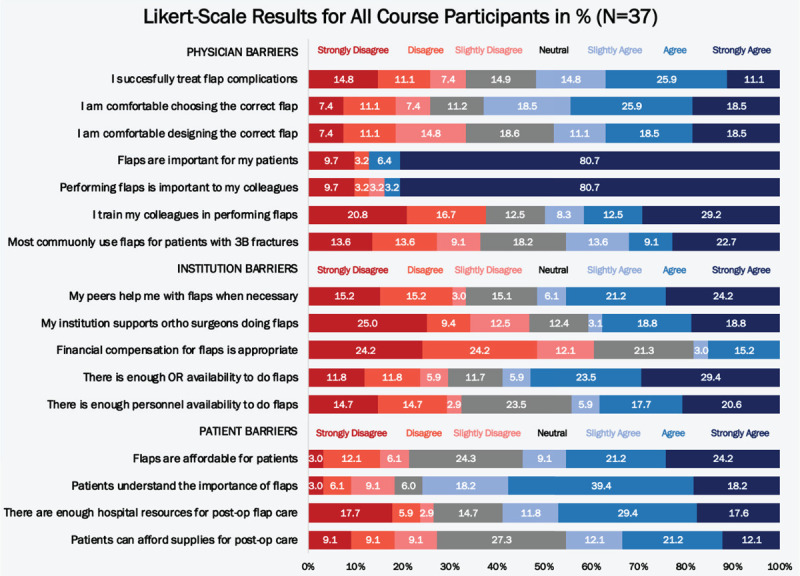
Participant response rate to Likert-scale barriers survey questions based on percentage of responses among overall course group.

Nearly half of all participants did not agree that they successfully treat flap complications (48.2%), and the majority did not agree that they felt comfortable designing the correct flap for a patient in need of soft-tissue coverage (51.9%). Similarly, more than half of participants did not agree that they commonly use flaps for Gustilo-Anderson Type 3B open tibia fractures (54.5%). Participants consistently found that institutional issues were significant barriers to performing flaps, with numerous participants either disagreeing or strongly disagreeing that their institution supports orthopaedic surgeons performing flap procedures (34.4%). Similarly, many either disagreed or strongly disagreed to feeling appropriately compensated for performing flaps (48.4%) and having enough operating room (OR) time (23.6%) and surgical personnel availability (29.4%). A number of participants also disagreed or strongly disagreed that their hospital had enough resources for postoperative flap care (23.6%) (Fig. 2).

There were no significant differences found regarding participant attitudes toward any barriers between participants with prior flap training versus those without. Participants’ confidence in designing a flap was significantly lower among participants stating that flaps were uncommonly completed at their hospital (3.8 vs. 5.3, *P* = .050). Availability of dermatomes was also reported to be lower in this group (12.5% vs. 57.1%, *P* = .010), and greater numbers of these participants reported that they lack peer support (3.3 vs. 6.0, *P* < .001), institutional support (3.2 vs. 4.9, *P* = .038), operating room availability (3.9 vs. 6.1, *P* = .002), and surgical personnel availability (3.3 vs. 5.7, *P* = .029). Lastly, this group reported lack of sufficient hospital resources for postoperative flap care (3.9 vs. 5.5, *P* = .029 (Table 2). Those with inadequate prior flap training reported feeling less confident in treating flap complications (4.1 vs. 6.0, *P* = .027), choosing the correct flap (4.2 vs 6.2, *P* = .022), and correctly designing a flap (4.0 vs. 6.0, *P* = .0141). They also reported less common use of soft-tissue coverage/flaps for Gustilo-Anderson Classification type IIIB fractures (3.4 vs. 5.8, *P* = .010) (Table 2).

**Table 2:**
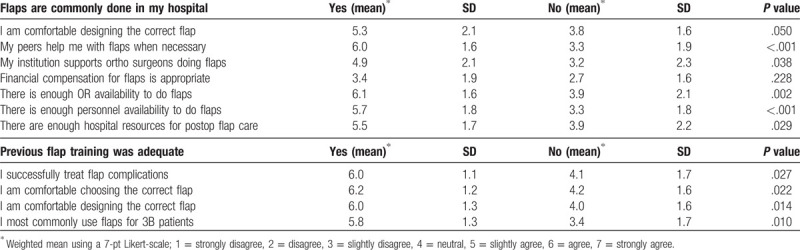
Subgroup analysis of barriers to soft-tissue coverage procedures

## 4 Discussion

In this study, we report the results of a survey of Latin American participants attending an orthopaedic and soft tissue-coverage procedure surgical training course in Mexico. Among attendees, there was improved immediate postcourse knowledge of soft tissue coverage procedures, particularly pertaining to plastic surgery. We identified several barriers to performing flaps including lack of plastic surgeon availability, inadequate training, lack of dermatome access, poor institutional and peer support, and constrained hospital resources for postoperative flap care.

Our findings are consistent with prior literature from lower resourced environments, demonstrating a lack of personnel with surgical technical expertise, requisite equipment, and institutional support in order to perform soft-tissue coverage procedures. A recent systematic review identified that key barriers to surgical care included lack of local resources, surgical expertise, and costs related to care.^[7]^ More specific to plastic surgery, a study from Nepal similarly identified a lack of plastic surgery surgical equipment, surgical specialists, and necessary structure and training for plastic surgeons.^[8]^ A similar survey study from Vietnam found that respondents reported a lack of surgical supplies, sufficient training, and prohibitive treatment costs.^[19]^ Finally, physicians in South Africa, an upper-middle income country, note that they, too, do not have enough plastic surgeons to meet the increasingly large amount of lower extremity trauma. The report from South Africa advocates for training orthopaedists in soft-tissue coverage procedures in settings where it may be challenging to rapidly scale the plastic surgery workforce.^[11]^

Orthopaedic training programs in LMICs have already been noted in the literature to be cost-effective measures for treating the large volume of musculoskeletal trauma. Grimes et al found that an orthopaedic clinical officer training program in Malawi cost $92 per DALY averted, which is dramatically less costly than other common global health interventions such as antiretroviral therapy for HIV or Malaria.^[21–23]^ Similarly, in Haiti, a training program for orthopaedic fellows had a cost of only $133.97 per DALY averted.^[24]^ It stands to reason that training orthopaedic surgeons in performing flap procedures in LMICs may be similarly cost-effective as other plastic surgery interventions. For example, across various LMICs, cleft lip and palate surgery costs from as little as $15 to $96 per DALY averted.^[25,26]^

The SMART course model is potentially efficacious in scaling up orthopaedic surgeon skills in performing flaps.^[4,15]^ These courses may have an additional knock-on effect by which surgeons will gain the skills to not only perform flap procedures, but also to teach such procedures to their peers. As many of the participants felt they lacked peer and institutional support to perform flap procedures, the SMART course may be a potential way to address these barriers. Participants may be able to act as local champions for flap completion at their institution by utilizing their new skills and providing evidence that flaps are feasible in resource limited settings.^[27–31]^

This study has several limitations. The knowledge surveys are an unvalidated tool for evaluating flap knowledge, and they do not assess long-term knowledge acquisition and retention. A longer follow-up period to assess knowledge retention would be valuable for assessing long-term course efficacy. The knowledge surveys were also not individually identifiable to maintain participant confidentiality. This resulted in unequal participation in the pre- and postcourse surveys, which affected the type of statistical analysis employed. However, the results are consistent with prior SMART course data demonstrating efficacy in improving postcourse knowledge survey scores.^[15]^ This study has a small sample size, and its findings may not be generalizable. Course attendees may have been more likely to attend given their lack of flap training thus identifying barriers that may not be perceived by surgeons at large. Last, the barriers survey is an unvalidated instrument, and it may not capture all barriers to performing flaps among this cohort.

Nonetheless, this is the first study we are aware of to report on the barriers to performing soft tissue coverage procedures among Latin American surgeons. We identify that many surgeons receive inadequate flap training and that a surgical skills training course may be an effective way to improve knowledge of these procedures. Furthermore, we identify cost-effective ways to overcome local barriers including increasing dermatome access, improving institutional and peer support, and providing adequate resources for postoperative flap care. In addressing these barriers, Mexican orthopaedic and plastic surgeons may begin reducing the morbidity of patients with severe lower extremity traumatic injuries.

## Acknowledgments

The authors thank the members of the board of FEMECOT for supporting the inaugural SMART course in Guadalajara, Mexico.
